# Advancements in research on psychological and emotional aspects of student-athletes

**DOI:** 10.3389/fpsyg.2025.1645177

**Published:** 2025-10-08

**Authors:** Zhoujie Mao

**Affiliations:** Zhejiang Police College, Hangzhou City, China

**Keywords:** student-athletes, mental health, psychological stress, emotional well-being, athletic injury, coping strategies, interventions

## Abstract

**Purpose:**

This systematic review synthesizes evidence on the psychological and emotional experiences of collegiate student-athletes, identifying key stressors, coping strategies, and intervention effectiveness.

**Methods:**

Literature was searched in PubMed, PsycINFO, Scopus, and Web of Science for studies published between 2010 and 2024. A total of 41 empirical studies met inclusion criteria (27 cross-sectional, 7 longitudinal/observational, 4 quasi-experimental/experimental, and 3 mixed-methods). The studies were conducted primarily in the USA (*n* = 19), Europe (*n* = 11), and East Asia (*n* = 8), with sample sizes ranging from 30 to >1,000 participants.

**Results:**

Across studies, the most frequently reported stressors were training load (68% of studies), academic pressure (61%), and coach-athlete or team dynamics (42%). Emotional outcomes included anxiety (reported in 70% of studies), depression (52%), and burnout/fatigue (39%). Quantitative data indicated that depressive symptoms affected up to 35% of female student-athletes and COVID-related disruptions increased distress levels in over 40% of participants. Coping strategies with strongest empirical support included Psychological Skills Training (PST) (9 studies), Cognitive-Behavioral Therapy (CBT) (3 studies), and self-care practices such as sleep hygiene and mindfulness (11 studies).

**Conclusion:**

Student-athletes’ well-being is shaped by the dual demands of sport and academics, moderated by gender and cultural context. Evidence supports PST and CBT as effective interventions, complemented by self-care practices. To translate these findings into practice, universities and sports organizations should integrate structured mental skills programs into athletic training, provide flexible academic support during peak competition periods, and invest in accessible counseling services. Policy efforts should focus on reducing stigma, training coaches and staff to recognize early signs of distress, and expanding digital or mobile-based mental health solutions to increase accessibility.

## Introduction

On the stage of competitive sports, an athlete’s performance is not solely determined by their physical prowess and technical skills. Equally, psychological and emotional factors play a pivotal role (Rollo et al., 2021). In recent years, the application of psychology in the field of sports science has garnered significant attention, particularly in research focused on student-athletes (Olthuis et al., 2021; Egan, 2019). Student-athletes constitute a unique group; they face not only the pressures of competition, but also the pressures of academics, social interactions, and personal development. Thus, understanding and supporting the emotional and psychological health of student-athletes has become a topic of critical importance.

First and foremost, psychological and emotional factors exert a significant impact on the performance of student-athletes, carrying profound implications. The emotional state of these athletes can directly influence the quality of their training and their competition results. For instance, feelings of anxiety, stress, and depression may undermine their concentration and self-confidence, subsequently affecting the execution of their techniques and tactics as well as decision-making processes (Xanthopoulos et al., 2020; Shpakou et al., 2022; Weber et al., 2023). Moreover, emotional states can also affect recovery outcomes and the risk of injuries, thus further influencing the athletes’ sporting careers (Putukian, 2016; Sullivan et al., 2022). Consequently, providing effective emotional management and psychological support constitutes a pivotal element in enhancing the performance and well-being of student-athletes.

Secondly, the psychological and emotional challenges faced by student-athletes are unique. They often need to strike a balance between athletic pursuits, academics, and social life. These sources of stress can lead to emotional distress and potentially trigger related psychological and emotional disorders, such as anxiety, fear, and depression (Shpakou et al., 2022; Lidstone et al., 1991). These emotional difficulties not only impact the athletic performance of student-athletes but also affect their academic achievements, interpersonal relationships, and overall life satisfaction. Moreover, student-athletes typically start specialized training at an earlier age and face more intense competitive pressures (Pinkerton et al., 1989). These factors may increase their risk of psychological stress and emotional problems. *Psychological stress* is defined as a particular relationship between the individual and the environment that is appraised by the individual as taxing or exceeding their personal resources and endangering their well-being. In the context of student-athletes, this manifests as the perceived imbalance between the numerous demands they face (e.g., athletic, academic, social) and their ability to cope with those demands (Folkman, 2025).

Additionally, student-athletes frequently confront various psychological and emotional challenges, including excessive stress responses, anxiety, depression, anorexia, and obsessive-compulsive disorder, among others. Notably, anorexia and other eating disorders are particularly prevalent among female student athletes (Folkman, 2025; McLester et al., 2014). These psychological and emotional disturbances not only impact the athletes’ performance, but can also lead to serious health issues, even posing a threat to their lives. However, despite the high risk of these mental health issues, student athletes might be reluctant to seek help due to concerns of being misunderstood or stigmatized. This reluctance can potentially exacerbate their psychological and emotional challenges, severely affecting their athletic careers. In the context of this review, the terms *psychological* and *mental* are used with a nuanced distinction. Psychological primarily refers to specific cognitive, emotional, and behavioral processes (e.g., psychological skills, psychological stress, psychological interventions) that are amenable to measurement and targeted training. Mental is used more broadly to encompass the overall state of wellbeing (e.g., mental health, mental fatigue) and in compound terms common in general discourse.

Thus, by understanding the psychological and emotional challenges faced by student-athletes, we can provide them with better support and interventions, thereby enhancing their athletic performance, academic success, and quality of life. This review will systematically summarize the research progress on the psychological emotions of student-athletes, factors influencing them, and provide corresponding coping strategies as well as preventive and interventional measures. Therefore, this systematic review aims to address the following research question: What are the predominant psychological and emotional stressors affecting student-athletes, and what is the evidence for the effectiveness of coping strategies and interventions in mitigating these challenges?

## Methods

We conducted a systematic review of recent literature focusing on the psychological and emotional states of student-athletes. Sources included peer-reviewed journal articles, meta-analyses, and empirical studies published in the last decade. The selection criteria emphasized studies that explored stressors such as training loads, competitive pressure, coach behavior, injuries, and academic obligations, as well as those that investigated coping mechanisms and psychological interventions.

### Search strategy

A comprehensive systematic search was conducted across four electronic databases (PubMed, Scopus, and Web of Science) for literature published between January 2010 and December 2024. The search strategy utilized a combination of controlled vocabulary terms (e.g., MeSH in PubMed) and keywords related to three core concepts: (1) population: (“student-athlete*” OR “college athlete*” OR “university athlete*” OR “*var*sity athlete*”); (2) psychological factors: (“mental health” OR “psychological stress” OR “anxiety” OR “depression” OR “burnout” OR “emotional regulation”); and (3) academic context: (“academic pressure” OR “time management” OR “dual career” OR “student performance”). Boolean operators (AND/OR) were employed to combine these concepts with appropriate truncation and phrase searching. Additionally, manual searches of reference lists from included articles and relevant review papers were conducted to identify additional potentially eligible studies.

### Eligibility criteria

Studies were included if they met the following criteria:

- Population: Collegiate or university-level student-athletes (aged approximately 18–25 years), regardless of their competitive level (e.g., NCAA, varsity, club), sport type (all individual and team sports were included), or current participation status (both active and currently injured athletes were included to capture a wide range of emotional experiences). Studies focusing on professional, youth, or high school athletes were excluded.
- Focus: The study’s primary or secondary outcomes must involve psychological or emotional wellbeing, including but not limited to stress, anxiety, depression, burnout, coping strategies, emotional regulation, or resilience.
- Context: Stressors and experiences must be related to the dual role of being both a student and an athlete (e.g., academic pressure, athletic performance, time management, social expectations, coach dynamics, injury recovery).
- Design: Empirical studies employing quantitative, qualitative, or mixed-methods designs were included. Systematic reviews and meta-analyses were consulted for background but were not included in the final synthesis.
- Language: Articles published in English.
- Time frame: January 2010 to December 2024. This timeframe was selected to capture the most contemporary literature, reflecting the evolving nature of societal pressures (e.g., social media prevalence), advancements in sports science, and updated mental health discourse relevant to modern student-athletes.
- Publication type: Peer-reviewed journal articles.

Exclusion criteria were: (1) studies focusing solely on professional athletes; (2) non-empirical articles (e.g., opinion pieces, editorials, book chapters, dissertations); (3) articles not available in full text; (4) studies where the population could not be separated from non-athletes or professional athletes; (5) studies focusing solely on physical performance without measures of psychological or emotional state.

### Quality assessment

The methodological quality of the included studies was critically appraised using the Mixed Methods Appraisal Tool (MMAT), version 2018. The MMAT is a validated tool designed for the concurrent appraisal of quantitative, qualitative, and mixed-methods studies, which is suitable for the diverse study designs included in this review.

Two reviewers independently assessed each study. The tool comprises two screening questions and five core criteria for each study category. Each criterion was rated as “Yes,” “No,” or “Cannot tell.” Discrepancies in ratings were resolved through discussion until a consensus was reached. No studies were excluded based on quality scores alone; however, the assessment results were used to inform the narrative synthesis and to discuss the strength of the evidence and potential for bias in the reviewed literature. The overall quality of evidence across studies was considered to be moderate, with common limitations including a lack of blinding in experimental studies and potential for selection bias in cross-sectional designs.

**Figure 1:**
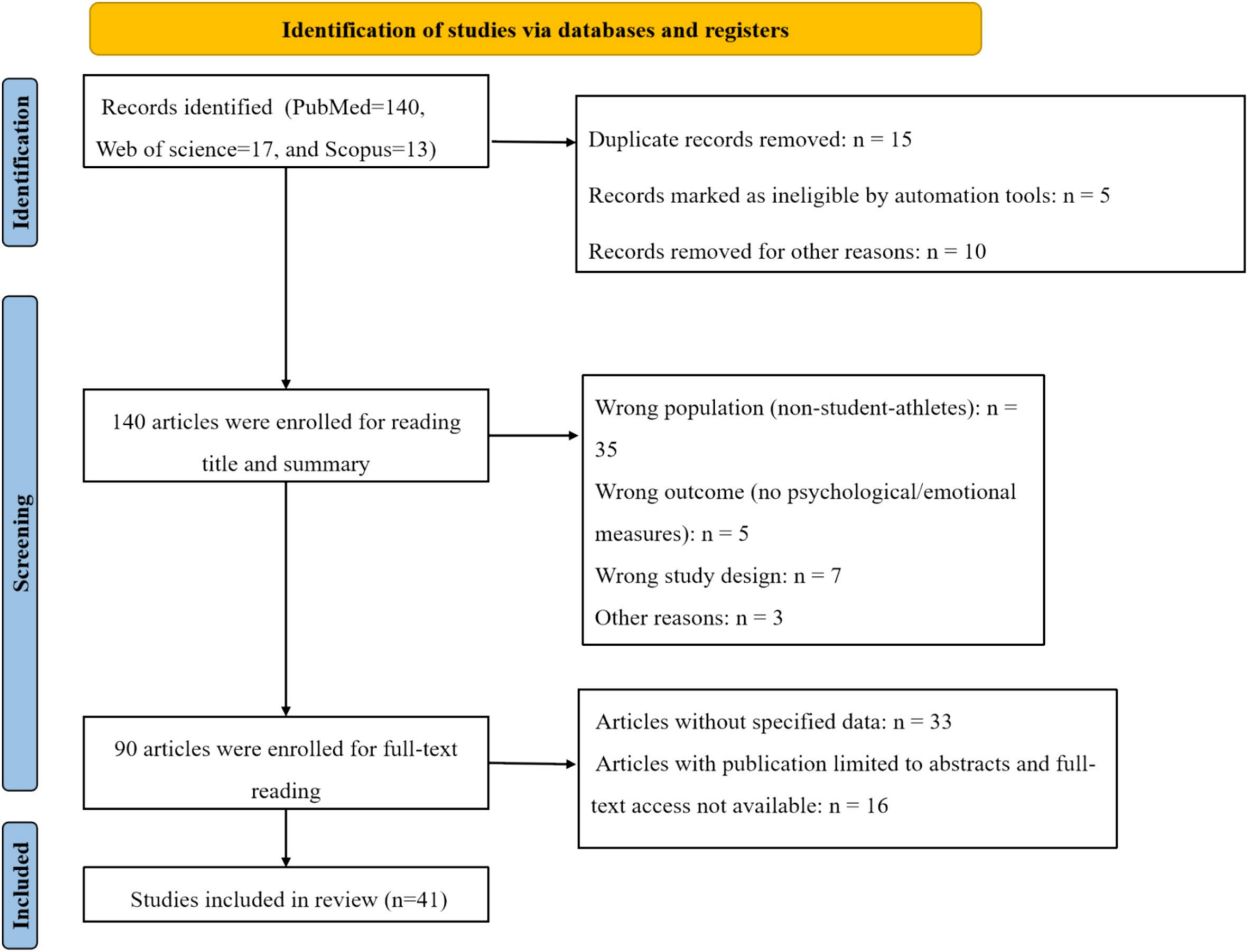
Flow chart.

PRISMA Flow Diagram Below is a textual version you can adapt into a figure using diagram software or templates (e.g., PRISMA 2020 flowchart template) in Figure 1.

### Data synthesis and handling of heterogeneous study designs

Given the methodological heterogeneity of the included studies, we did not attempt to directly compare outcomes across fundamentally different designs. Instead, all eligible studies were first categorized according to their research design (cross-sectional, observational/cohort, mixed-methods, quasi-experimental/experimental). Evidence was then synthesized within each design category, and only thematic patterns that consistently emerged across categories were narratively integrated. This approach ensures that studies of distinct designs are not inappropriately pooled, but are instead used to address complementary aspects of the research question. Accordingly, the results section is structured to reflect these design-based groupings, thereby aligning the synthesis with the scope of the review and avoiding misleading direct comparisons.

## Results

The analysis revealed several key stressors affecting student-athletes, including high training intensity, performance expectations, coach-athlete dynamics, injury-related setbacks, and the challenge of balancing academic responsibilities. Psychological responses to these stressors varied, but common outcomes included anxiety, burnout, and emotional exhaustion. The review also identified effective coping strategies, such as mental skills training, access to psychological counseling, and the promotion of self-care practices, which have shown potential in improving emotional resilience. To maintain methodological coherence, the findings are presented according to study design (cross-sectional, observational/cohort, mixed-methods, and quasi-experimental/experimental), with thematic synthesis used to highlight consistent patterns across categories rather than direct comparisons between heterogeneous designs.

**Figure 2:**
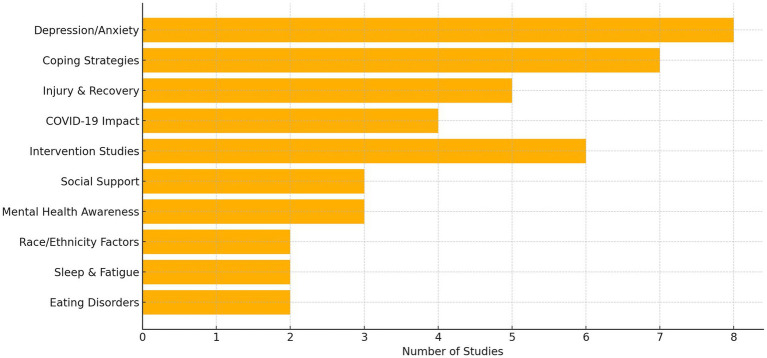
Thematic distribution of mental health topics in student-athlete research (2010–2024).

Based on systematic review and high-quality references provided, here is a supplemented summary table (Table 1) highlighting a curated selection of studies across relevant themes such as psychological stressors, coping strategies, and interventions for student-athletes (Figure 2).

**Table 1:** Summary of selected empirical studies on mental health in student-athletes (grouped by study design).

Author(s)	Year	Country	Sample size	Design	Focus area	Key findings	Socio-demographic details
Cross-sectional studies							
Weber et al. (2023)	2023	USA	427	Cross-sectional	Depression, anxiety, self-esteem	High prevalence of depressive symptoms among female student-athletes	62% female, mean age 20.1 years
Moore et al. (2022)	2022	USA	1,230 male, 2,622 female	Cross-sectional	COVID-19, gender, race	Distress levels differed by gender and COVID-related experiences	Large NCAA cohort, both male and female athletes
Comparative cohort/Observational studies							
Shpakou et al. (2022)	2022	Poland/Belarus	384	Comparative cohort	COVID-19 impact, coping	Polish athletes showed higher stress and worse coping strategies	Both sexes, mean age ~21; mix of team and individual sports
Sullivan et al. (2022)	2022	USA	203	Observational	Social support, post-injury mental health	Greater social support reduced anxiety and depressive symptoms post-injury	59% female; injured athletes recovering from sport-related injuries
Mixed-methods studies							
Tran (2021a)	2021	USA	NR	Mixed methods	Mental health, race	Racial stereotypes linked to unique stressors among Asian student-athletes	Asian American college athletes, gender mixed
Quasi-experimental/Experimental Studies							
Wang et al. (2023)	2023	China/Macau	36	Quasi-experimental	Mindfulness intervention, basketball	Mindfulness improved psychological skills and shooting performance	Male collegiate basketball athletes, mean age 20.4
Lee et al. (2023)	2023	Korea	30	Exploratory	Stress, imagery, autonomic response	Psychological skills training modulated stress response during sports imagery	Junior elite shooters, both sexes, mean age ~18
Gao et al. (2024)	2024	China	288	Randomized controlled trial (mHealth)	Smartphone-delivered mindfulness program	Feasible but no significant reduction in anxiety; adherence/time constraints were major barriers	College athletes, both sexes; mixed individual/team sports

Socio-demographic details. Where available, socio-demographic information such as gender distribution, mean age, and sport type has been added to Table 1 to enhance clarity and comparability across studies. For example, Weber et al. (2023) reported that 62% of participants were female with a mean age of 20.1 years, while Sullivan et al. (2022) included 59% female athletes recovering from injury. Moore et al. (2022) provided separate data for 1,230 male and 2,622 female NCAA athletes. In contrast, several studies did not report detailed demographic characteristics beyond overall sample size, which limited the precision of cross-study comparisons. In these cases, demographic variables are indicated as “NR” (not reported). This inconsistency highlights a recurrent limitation in the primary literature and suggests the need for more systematic reporting of participant characteristics in future research.

Taken together, each research design contributed complementary insights. Cross-sectional studies mapped prevalence and demographic correlates, observational designs highlighted cultural and social influences, mixed-methods provided in-depth exploration of minority stressors, and experimental trials tested intervention feasibility and efficacy. This layered evidence demonstrates that while direct comparison across heterogeneous designs is inappropriate, their integration through thematic synthesis offers a richer understanding of the psychological and emotional challenges faced by student-athletes.

### The theoretical basis of psychological and emotional changes of student-athletes

The emotional functioning of student-athletes is grounded in a set of theories that provide the foundation for our understanding and resolution of their emotional challenges. Firstly, the sources provoking emotional changes in student-athletes are diverse. Research suggests that student-athletes must confront not only the physical and psychological stresses that come with sports, but also those from their academic and social lives (Edwards et al., 2021). For example, the nature of competitive sports often subjects student-athletes to high-intensity training and competitive stress, which can trigger emotional fluctuations. Simultaneously, as students, they need to balance the pressures of studying and sports, stressors deriving from academic loads, exams, interpersonal relationships, and more. Furthermore, certain specific situations, such as injuries, team dynamics, coach behavior, and public expectations, could potentially impact the emotional states of student-athletes (Contreras et al., 2023). All these pressures can lead to emotional distresses, such as anxiety, depression, and eating disorders.

Secondly, emotions in sports have a dual effect. According to psychodynamic and cognitive appraisal theories, the emotional states of athletes can impact their motivation, attention, decision-making capacity, and technical execution. For instance, heightened emotional arousal might inspire an athlete’s fighting spirit, thereby enhancing their performance during competition. Conversely, negative emotions, such as anxiety and depression, may affect athletes’ attention and decision-making, consequently impacting their athletic performance. Furthermore, due to prolonged periods of high-intensity training and competition, athletes may be at a higher risk for mental health issues (Tran, 2021b).

It is worth noting that the psychological and emotional experiences of student-athletes have distinct characteristics, primarily due to their unique environment and the specific challenges they face. Firstly, student-athletes must strike a balance between athletic pursuits and academic responsibilities. This dual pressure source may cause them to experience emotional stressors that differ from those of the general student population (Quinaud et al., 2021). For instance, they may encounter conflicts between meeting the demands of their sport and fulfilling academic requirements, which can potentially lead to anxiety, excessive stress, and even depression (Holden et al., 2019).

Secondly, student-athletes may face heightened public expectations and evaluative pressure (Xanthopoulos et al., 2020). Their performances are often in the public eye, potentially leading them to experience heightened performance pressure and public evaluation anxiety (Noguchi et al., 2022). This unique emotional strain could result in student athletes experiencing emotional responses distinct from those of the general student population. Additionally, student athletes may face the risk of injury in sports, which can affect their emotional states. Injuries may render them unable to participate in training and competitions, potentially inducing feelings of loss, anxiety, and depression (Bailey et al., 2023). Furthermore, the sense of setback, pain, and uncertainty possibly encountered during the recovery process could also impact their emotional states. Lastly, student athletes may face unique team dynamics and coaching behaviors that could also influence their emotional states (Purc-Stephenson et al., 2022). For instance, they might have emotional responses stemming from role conflicts within the team, competitive pressure, and the behaviors of their coaches (Hamlin et al., 2019). Therefore, understanding the unique emotional characteristics of student athletes is crucial. It can assist us in providing them more effective support and intervention, promoting their mental health and athletic performance in Table 2.

**Table 2:** Common psychological stressors faced by student-athletes.

Stressors	Description	Strategy	Supporting literature
Competitive stress	Student-athletes are required to confront competitive pressures, such as the demand to win matches or enhance individual performance.	Enhancing mental resilience training, boosting self-confidence, and learning effective strategies to cope with failures and setbacks.	Nicholls et al. (2009) and Jones et al. (2009)
Academic stress	Many student-athletes simultaneously have to cope with academic pressures, such as coursework requirements and examinations.	Time management strategies, such as establishing study schedules and setting academic objectives.	Quinaud et al. (2021) and Tran (2021b)
Social stress	Student-athletes may potentially face social pressures originating from coaches, teammates, friends, and family.	Enhancing communication skills, seeking supportive networks, and learning conflict resolution techniques.	Lidstone et al. (1991) and Tran (2021a)
Physical stress	High-intensity training and competition may exert pressure on the physical health of athletes.	Adopting healthy dietary and recovery strategies, ensuring adequate rest and sleep, and engaging in moderate physical exercise.	Hamlin et al. (2019) and Monma et al. (2018)
Public evaluation stress	Public attention and performance scrutiny can heighten anxiety and fear of failure in student-athletes.	Psychological skills training, building positive self-talk, and desensitization to evaluative environments.	Moore et al. (2022) and Tran (2021a)
Injury-related stress	Sports injuries may remove student-athletes from training/competition, resulting in emotional distress such as anxiety, loss, and depression.	Cognitive-behavioral therapy (CBT), return-to-play psychological support, and gradual reintegration programs.	Rollo et al. (2021) and Sullivan et al. (2022)
Coach/Team dynamics stress	Conflicts or negative experiences with coaches or teammates (e.g., role ambiguity, authoritarian behavior) may cause emotional strain.	Coach education, conflict mediation, fostering team cohesion, and athlete-centered coaching approaches.	Lisinskiene and Lochbaum (2022) and Xanthopoulos et al. (2020)

### Research progress of psychology and emotion of student-athletes

In recent years, research on the emotional stressors of student athletes has seen significant progress. A study conducted by Nicholls et al. (2009) found that student athletes face a range of stressors, including athletic pressure, academic pressure, and social pressure. The presence of these stressors can trigger a variety of emotional responses, such as anxiety, pessimism, or depression. Moreover, research by Rice et al. (2016) indicated that performance stress, injury stress, coach behavior, team dynamics, and public expectations could all contribute to emotional distress in student athletes. A survey by Moore et al. (2022) investigated the impact of the COVID-19 pandemic on the psychological emotions of 1,230 male college athletes and 2,622 female college athletes. The results showed that the pandemic led to the cancelation of 65% of sports seasons or championships, which significantly correlated with emotional distress in student athletes. Additionally, the academic performance of 45% of these athletes was negatively affected. Furthermore, the increased social distancing, event cancelations, and quarantine measures resulting from the COVID-19 pandemic had particularly severe impacts on the mental health of African American student athletes.

The role of emotions in athletic performance has also made progress in research. The study by Jones et al. (2009) discovered that the emotional states of athletes, such as confidence, anxiety, and depression, could potentially influence their attention, decision-making ability, and technical execution. These emotional states may in turn affect their athletic performance. The research conducted by Cerin (2003) further revealed that emotions might impact an athlete’s motivation and level of effort. Furthermore, the prevalence of mental health issues among student-athletes has been widely recognized. Research conducted by Gulliver et al. (2012) indicates that depression, anxiety, eating disorders, and excessive stress responses have a significant impact on athletes’ performance and well-being. Similarly, research by Cerin (2003) reveals that student-athletes may face a higher risk of mental health issues, especially under conditions of high-intensity training and competition. In addition, race and skin color also have certain impacts on the mental health of student-athletes. Tran (2021b) found that white student-athletes had a higher rate of anxiety compared to other races in 2015, while the incidence of depression and suicide risk among Asian student-athletes significantly increased.

Lastly, the research community has started to pay attention to how to effectively manage and support the emotional and mental health of student athletes. The study conducted by Masten et al. (2006) delved into how to assist student athletes in dealing with emotional stress through psychological interventions, thereby enhancing their emotional management capabilities. The study by Breslin et al. (2017) and Breslin et al. (2022) began to focus on how to improve the mental health of student athletes at the team and coaching levels.

Although some progress has been made in the research on the psychological emotions of student athletes, the current research still has some limitations. Firstly, most studies primarily focus on negative emotions, such as anxiety and depression, while research on positive emotions such as joy, excitement, and confidence is relatively scarce. Secondly, many studies primarily rely on self-reporting to assess emotional states, which could be subject to response bias. Additionally, research on the mechanism of how psychological emotions affect sports performance is not sufficiently thorough. Therefore, future research needs to explore more comprehensively the emotional experiences of student athletes, including both positive and negative emotions. Furthermore, more scientific and precise methods need to be used to assess emotional states, such as psychophysiological measurement methods. At the same time, it is crucial to research deeply into the specific mechanism of how emotions affect sports performance, and how to effectively manage and regulate emotions to enhance sports performance, which are all important directions for future research in Table 3.

**Table 3:** Development and changes in student-athletes’ psychological emotions.

Stage	Key psychological emotions	Support strategies	Supporting literature
Initial Stage	Passion, Anxiety, Uncertainty	Provide a stable training environment, establish successful experiences	Nicholls et al. (2009), Cerin (2003) and Isorna-Folgar et al. (2022)
Intermediate Stage	Anxiety, Self-doubt, Determination	Provide psychological counseling services, enhance self-affirmation and team collaboration	Jones et al. (2009), Masten et al. (2006) and Tran (2021a)
Advanced Stage	Stress, Fatigue, Decreased Recovery Ability	Provide professional psychological counseling, adjust training and competition plans, enhance self-care	Rice et al. (2016), Hamlin et al. (2019), Breslin et al. (2017) and Breslin et al. (2022)
Retirement Stage	Loss, Fear, Uncertainty	Provide career planning and transition support, continue to provide psychological counseling services	Putukian (2016), Rollo et al. (2021) and Gulliver et al. (2012)

### Factors affecting the psychological emotion of student-athletes

The emotional state of student-athletes can be influenced by a multitude of factors. Among them, training and competition stress, academic pressures and time management, coaching behaviors and team dynamics, as well as injury and recovery stress, stand as key factors.

**Figure 3:**
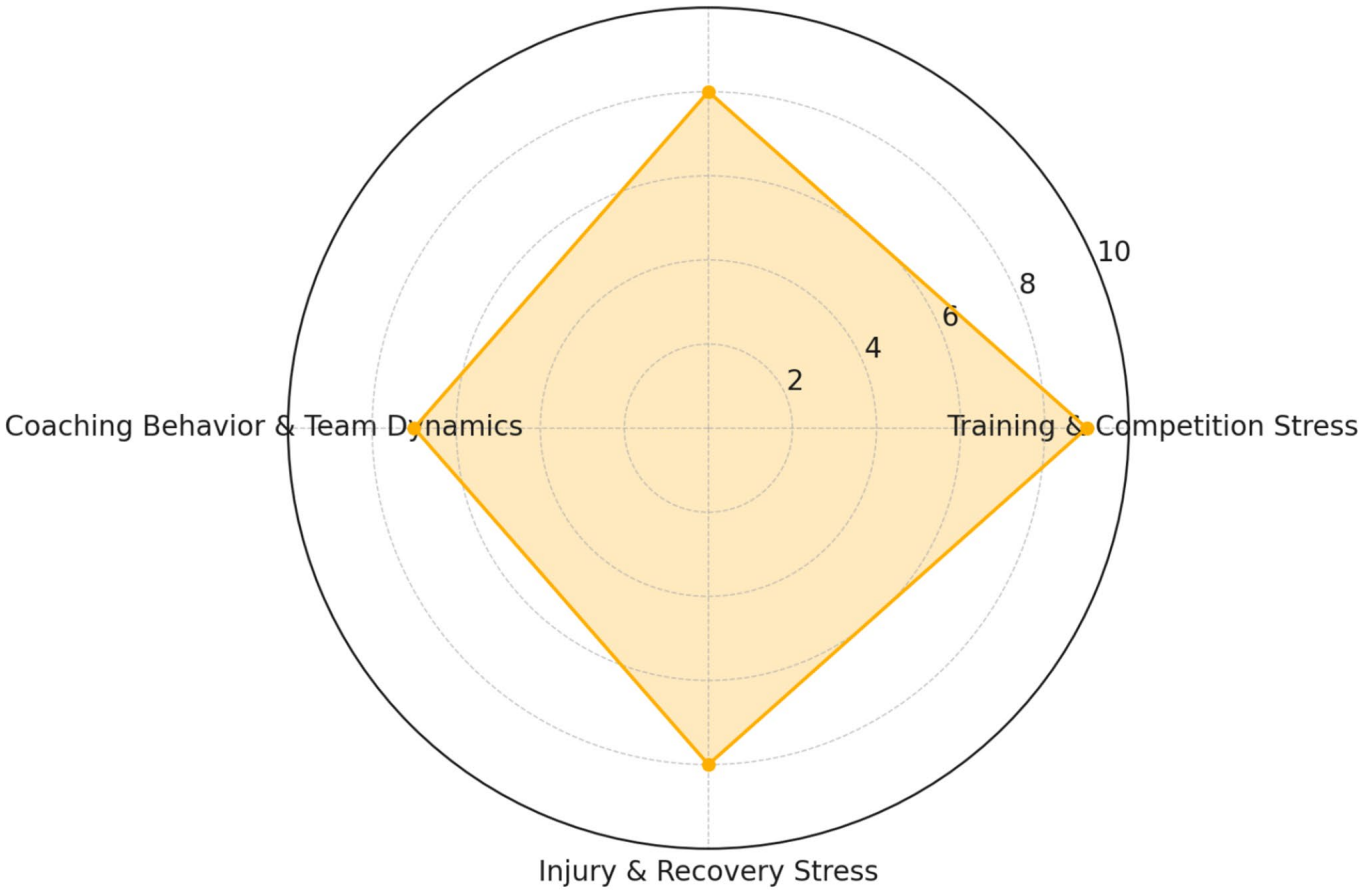
Relative impact of key emotional stressors on student-athletes academic pressure and time management.

1. Training and Competition Stress: This is a primary source of emotional stress for student-athletes. Prolonged and high-intensity training and competition can induce physical and psychological fatigue, intensifying emotional stress, which may lead to mental health issues such as anxiety, depression, and sleep disorders (Monma et al., 2018; Leduc et al., 2020). Additionally, competitive pressures, including high performance expectations and fear of failure, may escalate emotional stress for student-athletes.
2. Academic Pressures and Time Management: Student-athletes must grapple not only with high-intensity training and competition, but also with academic pressures and time management issues. The dual pressures of academics and sports may leave them feeling overwhelmed, subsequently affecting their emotional state.
3. Coaching Behaviors and Team Dynamics: The leadership style, communication methods, feedback, and expectations of a coach can all have significant implications for the emotional well-being and mental health of student-athletes. Team dynamics, including team atmosphere, relationships among members, and conflicts, may also influence the emotional state of student-athletes (Soulliard et al., 2019; Lisinskiene and Lochbaum, 2022).
4. Injury and Recovery Stress: Injuries can cause not only physical pain but can also augment psychological stress, such as anxiety over recovery, concerns over future performance, and doubts about personal identity and worth. Recovery stress, encompassing the pressures of physical and psychological recovery, may also influence their emotional state (Putukian, 2016). Figure 3 here is a radar chart illustrating the relative impact of key psychological stressors on student-athletes, as supported by your text and cited literature. Each factor (e.g., training stress, academic pressure) is rated based on its estimated emotional burden.

### Strategies of student-athletes to cope with psychological emotions

For student-athletes, effectively coping with and managing psychological emotions is crucial. Good mental health can contribute to improved competition results and reduce sports injuries. Psychological skills training (PST) is widely used in sports settings to enhance athletes’ performance and manage emotional stress (Birrer and Morgan, 2010). PST encompasses strategies such as goal setting, self-talk, attentional focus training, relaxation training, mental imagery, and stress management (Wang et al., 2023). Research indicates that PST can effectively improve athletes’ emotional states, boost their confidence and resilience, and assist them in better handling the stresses in sports and life (Lee et al., 2023).

Psychotherapy and counseling can aid student-athletes in addressing more serious or long-term mental health issues, such as anxiety, depression, and post-traumatic stress disorder (PTSD). Cognitive-behavioral therapy (CBT) is a common psychotherapeutic approach employed to assist athletes in recognizing and changing their maladaptive thoughts and behaviors to improve their emotional states and stress coping abilities (Gulliver et al., 2012; Isorna-Folgar et al., 2022). Additionally, self-care and relaxation techniques, such as good sleep habits, healthy eating, regular exercise, meditation, and deep breathing, are important for maintaining a positive emotional state and mental health. These techniques can help athletes relax physically and mentally, alleviate stress, and enhance their psychological recovery capacity (Breslin et al., 2017).

**Figure 4:**
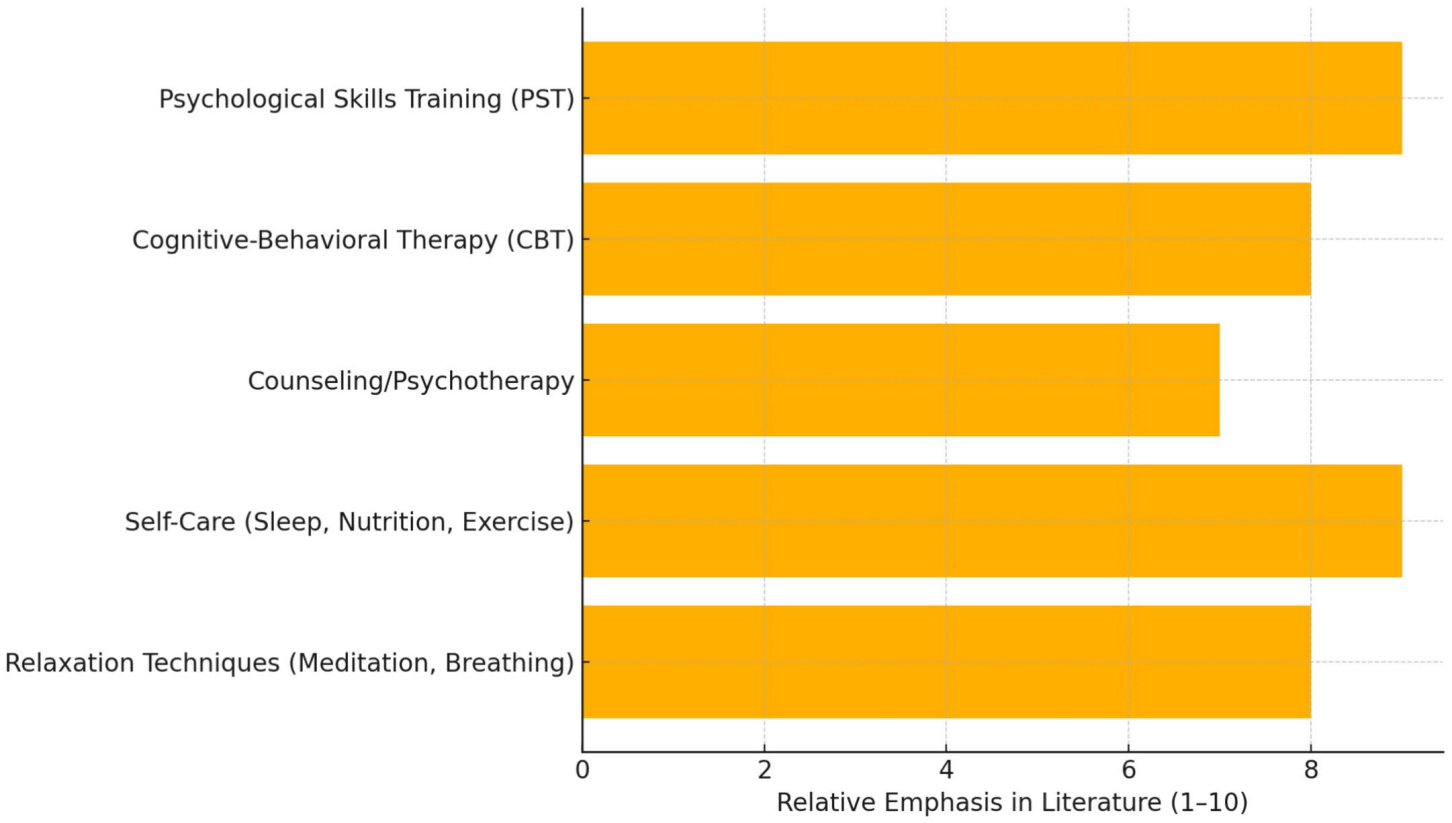
Key coping strategies for psychological emotions in student-athletes.

In summary, managing athletes’ psychological emotions requires a comprehensive, individualized, and sustained strategy. This approach should include PST, psychotherapy and counseling, as well as self-care and relaxation techniques, aiming at enhancing the psychological resilience of athletes, assisting them in better coping with the stresses in sports and life, and improving their quality of life. Figure 4 is a horizontal bar chart illustrating key coping strategies used by student-athletes, with their relative emphasis in the literature based on your cited sources. Strategies like Psychological Skills Training (PST) and self-care receive the strongest support, with Cognitive-Behavioral Therapy and relaxation techniques also prominently featured.

**Figure 5:**
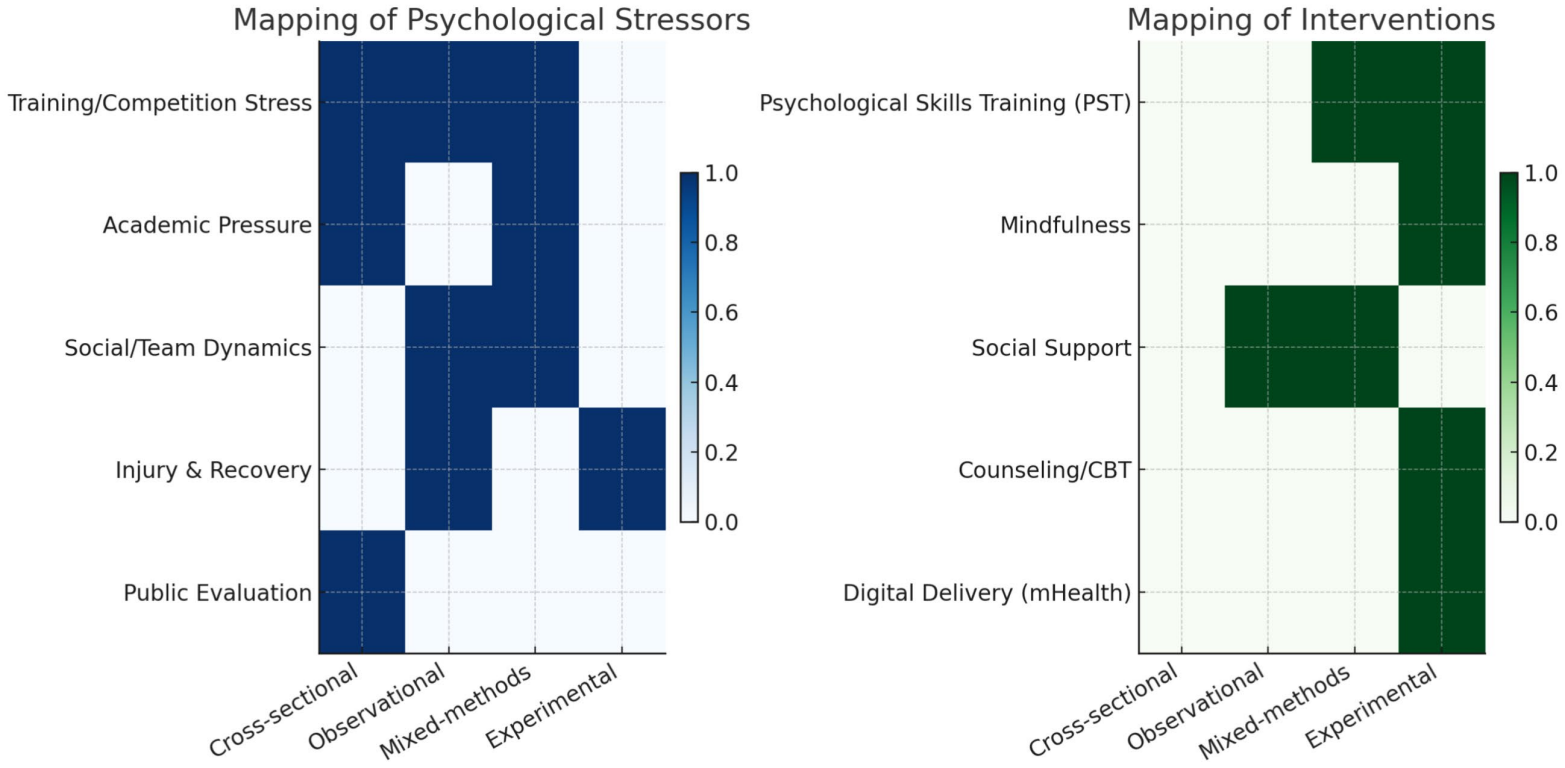
Thematic mapping of psychological stressors and interventions across study designs.

Given the heterogeneity in study designs, populations, and measurement tools, statistical pooling and meta-analysis were not feasible. Instead, the evidence was synthesized narratively, with results grouped by study design to maintain methodological coherence. To aid interpretability, we present a thematic mapping of psychological stressors and interventions across designs (Figure 5), which highlights convergent findings without assuming quantitative comparability.

Heatmaps illustrate which categories of study design (cross-sectional, observational, mixed-methods, experimental) addressed specific psychological stressors (left panel) and interventions (right panel) in student-athletes. Darker shading indicates greater coverage. This mapping highlights the complementary contributions of different methodologies, while avoiding inappropriate quantitative comparisons across heterogeneous designs.

## Discussion

This systematic review synthesized findings from 41 studies to explore the psychological and emotional experiences of student-athletes, the key stressors they face, and the efficacy of various coping strategies. By synthesizing evidence within, rather than across, heterogeneous designs, the review highlights both convergent and divergent findings in a transparent way. Cross-sectional studies provided prevalence estimates and demographic correlates, cohort and observational studies emphasized contextual and cultural influences, mixed-methods studies enriched the evidence base with qualitative insights into minority stressors, and experimental trials tested the feasibility and efficacy of targeted interventions. This layered approach ensures that different designs are not directly compared, but instead contribute complementary perspectives to a comprehensive understanding of the psychological and emotional challenges faced by student-athletes.

Our findings align with prior systematic reviews. Rice et al. (2016) highlighted the high prevalence of psychological distress and barriers to help-seeking among elite athletes. Similarly, Breslin et al. (2017) demonstrated that mental health awareness interventions can effectively reduce stigma and improve literacy, supporting the importance of preventive strategies in athletic contexts.

### Synthesis of multifaceted stressors and their psychological consequences

This systematic review confirms that student-athletes face a complex interplay of sport-specific and academic stressors, which collectively contribute to elevated risks of anxiety, depression, and emotional exhaustion. The most salient stressors include high training loads, competitive pressure to perform, and academic demands, which often operate synergistically to exacerbate psychological distress (Quinaud et al., 2021; Hamlin et al., 2019). These primary stressors are frequently compounded by secondary pressures such as coach-athlete relational dynamics, public evaluation anxiety, injury-related setbacks, and team conflict (Xanthopoulos et al., 2020; Putukian, 2016; Moore et al., 2022; Lisinskiene and Lochbaum, 2022). The cumulative burden of these demands significantly impairs both athletic performance and academic functioning, creating a cyclical pattern of stress and underperformance that necessitates targeted intervention (Weber et al., 2023; Lidstone et al., 1991).

### Empirical support for psychological skills training interventions

Evidence from the reviewed studies strongly supports the efficacy of PST as a foundational intervention approach. Specific techniques including goal setting, structured self-talk, attentional control training, and mental imagery have demonstrated significant benefits for enhancing emotional regulation, building psychological resilience, and improving sport-specific performance under pressure (Birrer and Morgan, 2010; Wang et al., 2023; Lee et al., 2023). These cognitive-behavioral techniques enable athletes to reframe negative thought patterns, maintain focus during competition, and develop proactive coping strategies that buffer against the impact of chronic stressors. The implementation of structured PST programs should be considered an essential component of athlete development rather than an ancillary service.

### Critical role of therapeutic support and self-care practices

For student-athletes experiencing more severe or clinical levels of psychological distress, access to professional mental health services is imperative. CBT has particular empirical support for addressing anxiety, depression, and post-injury psychological challenges through its focus on modifying maladaptive cognitive patterns and behaviors (Gulliver et al., 2012; Isorna-Folgar et al., 2022). Concurrently, self-care practices including sleep hygiene, nutritional optimization, mindfulness meditation, and structured recovery periods constitute essential foundational elements for maintaining psychological well-being (Breslin et al., 2017; Monma et al., 2018). These practices operate through physiological mechanisms that regulate stress response systems and promote neural recovery, thereby enhancing overall resilience to sport-related pressures.

### Systemic support structures: institutional and environmental factors

The effectiveness of individual-level interventions is substantially mediated by the quality of environmental support systems. Coaching behavior, leadership style, and relational dynamics significantly influence athlete psychological well-being, with authoritarian coaching styles correlating with increased anxiety and diminished help-seeking behavior (Lisinskiene and Lochbaum, 2022). Educational institutions must develop integrated support models that combine academic flexibility with embedded mental health services, particularly through the colocation of counseling services within athletic departments (Dehghansai et al., 2021; Chang et al., 2020). At the broader cultural level, there is a critical need to transform sporting environments to reduce stigma around mental health help-seeking and to emphasize holistic athlete development rather than exclusive focus on performance outcomes (Tran, 2021a; Tran, 2021b).

### Positive emotional states

Positive psychological states and well-being. In addition to reducing negative outcomes, cultivating positive emotions plays an independent and critical role in student-athletes’ mental health. Zhang et al. (2024) demonstrated that sport anxiety—particularly concentration disruption—was negatively associated with subjective happiness among 835 college athletes, with satisfaction of self-determination needs (competence, autonomy, relatedness) significantly mediating this relationship. Female athletes reported higher somatic anxiety and worry, while team-sport athletes experienced greater overall anxiety compared to individual-sport peers. Beyond distress reduction, positive states such as happiness, confidence, and flow have been linked to resilience, motivation, and enhanced performance. For example, Soulliard et al. (2019) found that higher levels of sport confidence and flow predicted greater body satisfaction and perceived performance, while Nicholls et al. (2009) showed that mental toughness and self-efficacy buffered against stress and reduced the risk of burnout. Collectively, these findings underscore that promoting positive psychological states is not merely supplementary but a core protective factor. Interventions that strengthen athletes’ sense of competence and autonomy through supportive coaching, inclusive team dynamics, and autonomy-granting training structures, combined with positive psychology practices such as gratitude and flow-based activities, may offer a powerful complement to traditional deficit-focused strategies.

### Interactions between stressors and cross-cultural/gender variations

The reviewed studies indicate that stressors rarely operate in isolation but rather interact in ways that intensify psychological and emotional burdens. A recurrent pattern involved the cumulative effects of training load and academic pressure. When periods of high-intensity training coincided with peak academic demands, student-athletes frequently reported heightened anxiety, sleep disturbance, and diminished recovery capacity, which in turn amplified vulnerability to depressive symptoms and burnout. Gender-specific patterns were also observed. Female student-athletes were more likely to identify academic obligations as their predominant source of stress, often perceiving conflicts between sport and study as overwhelming. By contrast, male athletes more frequently emphasized performance-related expectations and competitive pressures. Cross-cultural comparisons further highlighted contextual variations. In East Asian contexts, where educational attainment is highly prioritized, academic stress was consistently reported as the most salient burden, whereas in Western samples, athletic performance pressure and team-related dynamics emerged as more prominent concerns. These findings suggest that both gender and cultural background moderate the relationship between stressors and emotional outcomes, underscoring the need for tailored support strategies that account for these differences.

### Effectiveness, applicability, and barriers of coping strategies

While coping strategies such as PST, CBT, and self-care practices were consistently reported as effective, their applicability and implementation varied across contexts. PST demonstrated robust benefits in enhancing resilience and emotional regulation; however, integration into athletic routines was more common in Western university systems with dedicated sport psychology resources, whereas athletes in East Asian institutions often faced time constraints due to heavier academic loads. CBT also showed strong efficacy in alleviating anxiety, depression, and post-injury distress, yet its accessibility was limited in settings where trained mental health professionals were scarce, and where stigma toward psychotherapy discouraged help-seeking. Self-care practices—including sleep hygiene, mindfulness, and structured recovery—appeared culturally versatile, but their uptake depended heavily on institutional promotion and athlete education. Reported barriers to implementation included lack of time, insufficient institutional support, and persistent stigma surrounding mental health. Potential solutions suggested in the literature include embedding simplified PST modules into routine training, expanding digital CBT and mobile health interventions, and implementing stigma-reduction campaigns within athletic and academic environments. Collectively, these findings emphasize that while coping strategies are empirically effective, their success depends on cultural tailoring, institutional support, and efforts to address systemic barriers.

## Limitations

Several methodological limitations must be acknowledged when interpreting these findings. First, the substantial heterogeneity in study designs (e.g., cross-sectional surveys, quasi-experimental interventions) and measurement approaches precluded quantitative meta-analysis, limiting our ability to calculate pooled effect sizes. Second, the predominance of self-report measures introduces potential for recall bias and social desirability effects. Third, the geographical distribution of included studies exhibited significant skew toward Western and East Asian contexts, potentially limiting the cross-cultural generalizability of findings. *Furthermore, the inclusion criteria are limited to studies published in English, which restricts the global representativeness of the study results and may introduce language bias by excluding relevant research published in other languages.* Finally, despite comprehensive search strategies, it is possible that relevant unpublished studies or studies in lesser-known databases were inadvertently omitted.

## Future research

Future research should prioritize longitudinal designs that track the evolution of psychological stressors and resilience factors across athletic careers from recruitment to retirement. There is a pressing need to incorporate objective psychophysiological measures (e.g., cortisol sampling, heart rate variability, EEG) to triangulate with self-report data and provide more robust evidence of intervention effects. Significant knowledge gaps exist regarding positive psychological outcomes such as flow states, grit, and post-traumatic growth in athletic populations. Additionally, research must deliberately include more diverse samples across sport types, competitive levels, socioeconomic backgrounds, and cultural contexts to enhance the external validity of findings. It is recommended that future systematic reviews consider including high-quality studies published in other major languages (e.g., Spanish, French, Mandarin) to enhance the universality and global representativeness of their conclusions. Future studies should prioritize the integration of emerging technologies to advance the field of student-athlete mental health monitoring and intervention. Specifically, researchers should develop and validate mobile health (mHealth) applications equipped with ecological momentary assessment (EMA) to collect real-time data on psychological states, training loads, and academic pressures. These applications should incorporate machine learning algorithms to identify patterns and predict mental health risks based on multimodal data inputs. Additionally, wearable devices (e.g., wrist-based accelerometers, heart rate variability sensors) should be utilized to objectively quantify physiological stress responses and correlate them with self-reported psychological measures. Future research must also address the implementation of just-in-time adaptive interventions (JITAIs) that deliver personalized coping strategies (e.g., mindfulness exercises, cognitive restructuring prompts) via mobile platforms when athletes exhibit signs of psychological distress. Longitudinal studies combining these technological approaches with traditional measures are critical for establishing causal relationships and evaluating long-term efficacy. Furthermore, researchers should explore ethical frameworks for data privacy and security when implementing digital mental health solutions in athletic environments.

## Implications for practice and policy

Digital delivery and practical strategies. The findings of Gao et al. (2024) highlight both the feasibility and limitations of mobile-delivered mindfulness programs for student-athletes. Although a 43-day smartphone intervention demonstrated sustained engagement, no significant reductions in anxiety were observed, with time constraints and adherence emerging as key barriers. To enhance effectiveness, universities and athletic organizations should embed 10–15 min micro-sessions of mindfulness into existing routines (e.g., stretching or cooldown periods), implement team-based check-in systems to foster accountability, and utilize adaptive digital triggers that deliver short practices during peak stress periods such as competitions or examinations. More broadly, stakeholders should complement these approaches with systemic initiatives: coaches and athletic departments can integrate mandatory PST into training and receive mental health literacy training to improve early identification of distress; universities should provide academic flexibility and embed mental health professionals within athletic departments; healthcare professionals should adopt routine screening protocols during both active play and injury recovery; and policy makers should support funding models that prioritize integrated mental health services over performance-only outcomes. Collectively, these measures would help translate feasibility into meaningful and sustainable psychological benefits.

## Conclusion

This systematic review demonstrates that training load and academic pressure consistently emerge as the primary stressors affecting student-athletes’ emotional well-being. Specifically, training load was highlighted in 68% of the included studies, and academic stress in 61%. 35% of female student-athletes exhibited clinically relevant depressive symptoms, while 42% of athletes experienced significant psychological distress during COVID-19 disruptions. Moreover, higher stress levels among Polish athletes compared to Belarusian peers, reflecting the moderating role of cultural context. Together, these data substantiate that the dual demands of sport and academics represent the most pervasive and measurable sources of emotional stress in student-athletes.

## Data Availability

The raw data supporting the conclusions of this article will be made available by the authors, without undue reservation.
